# Staphylococcal Adhesion and Host Cell Invasion: Fibronectin-Binding and Other Mechanisms

**DOI:** 10.3389/fmicb.2017.02433

**Published:** 2017-12-05

**Authors:** Jérôme Josse, Frédéric Laurent, Alan Diot

**Affiliations:** ^1^International Center for Infectiology Research, INSERM U1111, CNRS UMR5308, ENS Lyon, Lyon 1 University, Lyon, France; ^2^Institute for Infectious Agents, Hôpital de la Croix-Rousse, Hospices Civils de Lyon, Lyon, France; ^3^French National Reference Centre for Staphylococci, Lyon, France; ^4^Microbiology-Mycology Department, Institut des Sciences Pharmaceutiques et Biologiques de Lyon, Lyon, France

**Keywords:** staphylococci, fibronectin-binding protein, adhesion, non-professional phagocytic cell, cell invasion

## Abstract

Opportunistic bacteria from the genus *Staphylococcus* can cause life-threatening infections such as pneumonia, endocarditis, bone and joint infections, and sepsis. This pathogenicity is closely related to their capacity to bind directly to the extracellular matrix or to host cells. Adhesion is indeed the first step in the formation of biofilm or the invasion of host cells, which protect the bacteria from the host immune system and facilitate chronic infection. Adhesion relies on the expression of a repertoire of surface proteins called adhesins, notably microbial surface components recognizing adhesive matrix molecules. In this short review, we discuss the main pathway (FnBP-Fn-α5β1 integrin), as well as alternatives, through which *Staphylococcus aureus* adheres to and then invades non-professional phagocytic cells. We then examine the corresponding mechanisms for coagulase negative staphylococci. There is currently a little understanding of the molecular mechanisms that lead to internalization. Filling this gap in the literature would therefore be an important step toward limiting the duration of staphylococci infections in clinical practice.

## Introduction

Staphylococci are commensal bacteria that make up a large part of the microbiota of skin and mucous membranes. In pathogenic conditions, they cause opportunistic and life-threatening infections such as pneumonia, endocarditis, bone and joint infections, and sepsis (Lowy, 1998; Becker et al., 2014). So far, 47 species and 23 sub-species of staphylococci have been identified, of which a few are coagulase-positive, such as *S. aureus* or *S. pseudintermedius*, but most are coagulase-negative species (CoNS), e.g., *S. epidermidis*, *S. lugdunensis, S. saprophyticus*, or *S. haemolyticus* (Becker et al., 2014). Like most bacteria, staphylococci express a broad range of surface proteins involved in their adhesion to extracellular matrix (ECM), plasma proteins or directly to host cells. The most prevalent of these proteins are the microbial surface component recognizing adhesive matrix molecules (MSCRAMMs), also found in enterococci and streptococci (Patti et al., 1994). All MSCRAMMs share a similar structure, with two adjacent IgG-folded domains mediating their attachment to components of the host ECM such as collagen, fibrinogen, or Fn (Becker et al., 2014; Foster et al., 2014). This binding capacity is closely related to the pathogenicity of staphylococci since their adherence to ECM or plasma proteins is a crucial step in the formation of biofilm and in the invasion of host cells (Löffler et al., 2014; Moormeier and Bayles, 2017). In this review, we discuss the mechanisms of cell adherence and internalization of coagulase-positive and -negative staphylococci. The main focus will be on the role of the ECM protein Fn and staphylococcal Fn-binding proteins (FnBPs) in the adhesion to and invasion of non-professional phagocytic cells (NPPCs) such as epithelial cells, endothelial cells, fibroblasts, and osteoblasts.

## *Staphylococcus aureus* Adhesion and Internalization by Host Cells

### The *FnBP-Fn-α5β1 Integrin* Pathway

The capacity of *S. aureus* to adhere to cells has been known since the early 1980s (Aly et al., 1980) and has been demonstrated for both primary cells and cell lines from various tissues (Ellington et al., 1999; Kerro Dego et al., 2002). Host cell adhesion mainly involves Fn forming a bridge between α5β1 integrin on the cellular side and Fn binding proteins (FnBPs, which are MSCRAMMs) on the bacteria (Tran Van Nhieu and Isberg, 1993; Sinha et al., 1999; Fowler et al., 2000; Grundmeier et al., 2004). This step is a prerequisite for any internalization into NPPCs; indeed, DU5883, an isogenic mutant of *S. aureus* NCTC 8325-4 defective in FnBP expression, cannot invade NPPCs (Fowler et al., 2000).

**FIGURE 1:**
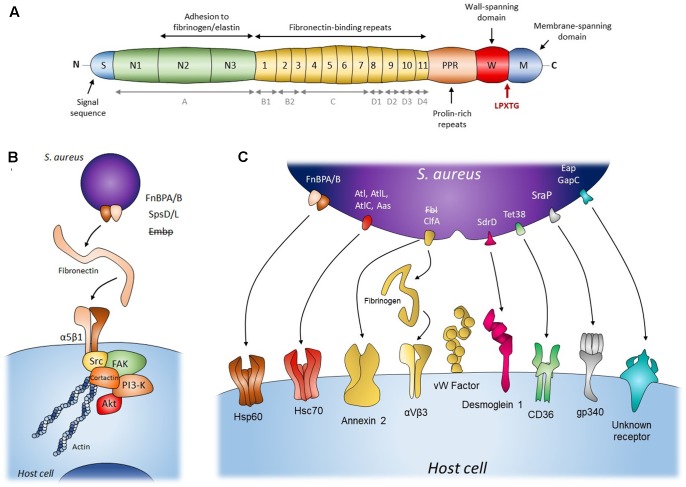
Staphylococcal mechanisms of adherence to and internalization into host cells. **(A)** Schematic diagram of structural organization of FnBP from *S. aureus*. Gray items (A, B1, B2, C, D1, D2, D3, D4) represent an alternative nomenclature to the fibronectin-binding repeats. **(B)** The Fibronectin α5β1 integrin pathway for adherence and internalization of *S. aureus* (FnBP A/B), *S. pseudintermedius* (SpsD/L) and *S. epidermidis* (Embp). This internalization pathway was hypothesized for *S. epidermidis* (Embp) but refuted. **(C)** Staphylococcal secondary mechanisms involved in adherence to and internalization into host cells. Bacterial adhesins presented in the figure’s panel are FnBP A/B, adhesion/autolysin family (Atl), fibrinogen binding adhesion (Fbl and ClfA), sdrD, Tet38, SraP, Eap, and GapC. The internalization pathway including Fbl, fibrinogen and host receptor was hypothesized for *S. lugdunensis* but refuted. Please refer to **Table 1** for adhesin/*Staphylococccus* species concordance.

There are two FnBP isoforms in *S. aureus*, FnBPA and FnBPB, encoded respectively by the *fnbA* and *fnbB* loci, with very similar domain organizations and sequences (Jönsson et al., 1991; Burke et al., 2010). However, their presence varies across the population (Peacock et al., 2000). They consist of an amino-terminal secretion signal sequence followed by an A domain that is closely related to fibrinogen-binding protein clumping factor A (ClfA, **Figure 1A**). This domain can bind fibrinogen and elastin and is involved in Fn binding (Wann et al., 2000; Roche et al., 2004; Burke et al., 2011). The A domains of FnBPA and FnBPB only share 40% sequence identity (Burke et al., 2010). The A domain is followed by tandem repeats of Fn-binding regions (FnBRs, 95% identity between FnBPA and FnBPB), 11 in FnBPA versus 10 in FnBPB (**Figure 1A**). This additional FnBR, along with the higher Fn affinity of certain FnBRs, might explain FnBPA’s higher overall affinity for Fn and the fact that FnBA alone is sufficient for adhesion and cell invasion (Grundmeier et al., 2004; Testoni et al., 2011). Finally, the C-terminal peptidoglycan-binding motif (LPXTG) and the wall and membrane spanning domains anchor FnBPs to the cell wall.

*In vivo*, the deletion of either FnBP strongly attenuates the ability of strain SH1000 to colonize the kidney and multiply, and to cause fatal sepsis in mice. Although FnBPA contributes more to these symptoms than FnBPB does, both FnBPs appear necessary for the development of severe infections (Shinji et al., 2011). This observation suggests that cooperation between the two FnBPs may be required for strong cell adhesion or to efficiently trigger the internalization pathway *in vivo*.

The high affinity and specificity of FnBPs for Fn is conferred by the tandem β-zipper structure they form together (Fowler et al., 2000; Schwarz-Linek et al., 2003). Fn bridges *S. aureus* and the host cell through its binding to cellular α5β1 integrin (Fowler et al., 2003). The efficiency of this adhesion varies both between cell types—one high-affinity FnBR is sufficient for adhesion and invasion in endothelial cells (Edwards et al., 2010) but three are needed in keratinocytes (Edwards et al., 2011)—and *S. aureus* strains. Indeed, whereas studies have shown that isolates from infections of cardiovascular devices have amino acid changes in the FnBR domain that increase the affinity for Fn (Hos et al., 2015), this is not the case for isolates from prosthetic joint infections (Eichenberger et al., 2015). Likewise, certain methicillin-resistant *S. aureus* strains involved in endovascular infections harbor an additional FnBR that, together with a substitution in FnBR 11, reduces its affinity for Fn but promotes cell invasion. Although this fits with the hypothesis that the pathogenesis of endovascular infections involves the invasion of endothelial cells, it seems to go against the idea that better adhesion promotes internalization. However, it is estimated that one FnBP can bind 6–9 Fn molecules (Bingham et al., 2008) and thus could cluster α5β1 integrins on the cell surface to trigger the efficient intracellular signaling required for internalization. One hypothesis is that when affinity for Fn decreases, more interactions are needed to achieve sufficient cell adhesion, mobilizing more Fn and α5β1 integrins and thus leading to their clustering.

The signaling pathway of staphylococci internalization involves focal adhesion kinases (FAKs) and activated Src (Fowler et al., 2003; Agerer et al., 2005) that subsequently recruit cortactin to promote actin polymerization and mobilize the endocytic machinery (**Figure 1B**) (Agerer et al., 2005; Selbach and Backert, 2005). Downstream of the FAK-Src pathway, the activation of PI3K and Akt is also important for the internalization of *S. aureus* (Oviedo-Boyso et al., 2011; Wang et al., 2013), although the molecular pathway is still far from being fully understood. Previous studies have shown that internalization is inhibited by cytochalasin D (Ellington et al., 1999; Sinha et al., 1999) and is temperature dependent (Sinha et al., 1999, specifically, inhibited at 4 and 14°C and facilitated at 37°C versus room temperature). These results respectively show that the biological prerequisites for internalization are (1) a dynamic actin cytoskeleton and (2) a fluid host cell membrane. Since bacterial uptake can occur with heat-killed or fixed bacteria, this mechanism appears to be an active process on the cellular side only. However, it has also been shown that *S. aureus* can stimulate its own uptake by upregulating β1 integrin expression in the host cell by secreting α-hemolysin. In this case, the bacteria must be viable as the process requires both cellular and bacterial activity (Abel et al., 2011; Goldmann et al., 2016).

In summary, *S. aureus* adhere to cells via interactions between FnBPs, Fn, and α5β1 integrins. The resulting clustering of integrins may then be sufficient to trigger the signaling cascade involving FAK, Src, PI3K, and Akt. Finally, *S. aureus* mobilizes the actin cytoskeleton and possibly the endocytosis machinery to enter host cells (**Figure 1**).

The FnBP-Fn-α5β1 integrin pathway is widely acknowledged to be the main internalization process. However, other factors have been shown to affect the efficiency of this internalization. In epithelial cells indeed, the internalization efficiency is maximal when the bacterial FnBPs interact directly with Hsp60 at the cell surface (Dziewanowska et al., 2000). Whether Hsp60 acts as a co-receptor to strengthen the binding of FnBPs-Fn to α5β1 integrin or is involved in signal transduction has still to be investigated (**Figure 1C**). Another clue that the FnBP-Fn-α5β1 integrin pathway is not the only internalization mechanism is that blocking α5β1 integrin or the binding of Fn by FnBPs with an antibody does not fully prevent internalization by MG63 osteoblast cells (Testoni et al., 2011) and by primary keratinocytes (Kintarak et al., 2004). The latter even internalize the FnBP-defective strain DU5883. Together, these results suggest that alternative mechanisms are involved in the adhesion and internalization of *S. aureus* (**Table 1**).

**Table 1:** Staphylococcal mechanisms of adherence to and internalization into host cells regarding of MSCRAMMs.

MSCRAMM	*Staphylococcus* sp.	Adherence to fibronectin	Internalization				Reference
			Capacity	Bridge	Host component	Host cell type	
FnBP A/FnBP B	*S. aureus*	Yes	High	Fibronectin	Integrin α5β1	Osteoblast, HEK-293	Tran Van Nhieu and Isberg, 1993; Sinha et al., 1999; Fowler et al., 2000
FnBP A/FnBP B	*S. aureus*	Yes	Low	No	Hsp60	Keratinocyte	Dziewanowska et al., 2000
SpsD/SpsL	*S. pseudintermedius*	Yes	High	Fibronectin	Integrin α5β1	Osteoblast	Pietrocola et al., 2015; Maali et al., 2016
Embp	*S. epiderrmdis*	Yes	No	(Fibronectin)	(Integrin α5βl)	Osteoblast	Khalil et al., 2007
Atl	*S. aureus*	Yes	?	Fibronectin ?	Integrin α5βl ?	Keratinocyte	Hirschhausen et al., 2010
Atl	*S. aureus*	Yes	Low	No	Hsc70	Keratinocyte	Hirschhausen et al., 2010
AltE	*S. epiderrmdis*	Yes	Low	No	Hsc70	Keratinocyte	Hirschhausen et al., 2010
AtL	*S. lugdunensis*	Yes	Low	No ?	Hsc70 ?	Epithelial cell, endothelial cell	Pereira et al., 2012; Hussain et al., 2015
Aas	*S. saprophyticus*	Yes	Low	No ?	Hsc70 ?	Hep2 cell	Hell et al., 1998, 2003; Szabados et al., 2008
AtlC	*S. caprae*	Yes	Low ?	No ?	Hsc70 ?	NR	Allignet et al., 1999, 2001, 2002
ClfA	*S. aureus*	Yes	Yes	No	Annexin 2	MAC-T cell	Ashraf et al., 2017
ClfA	*S. aureus*	Yes	NT	Fibrinogen	aVB3	Endothelial cell	McDonnell et al., 2016
ClfA	*S. aureus*	Yes	NT	vWbp	von Willebrand factor	Endothelial cell	Claes et al., 2017
Fbl	*S. lugdunensis*	Yes	No	(Fibrinogen)	(aVB3)	Endothelial cell	Szabados et al., 2011
SdrD	*S. aureus*	NT	Low	No	Desmoglein 1	Keratinocyte, nasal epithelial cell	Corrigan et al., 2009; Askarian et al., 2017
Tet38	*S. aureus*	NT	Low	No	CD36	A549 cell	Truong-Bolduc et al., 2017
SraP	*S. aureus*	NT	Low	NT	gp340	A549 cell	Yang et al., 2014
GapC	*S. aureus*	NT	Low	NT	NT	MAC-T cell	Kerro-Dego et al., 2012
Eap	*S. aureus*	NT	Low	NT	NT	Fibroblast	Palma et al., 1999; Hussain et al., 2002; Grundmeier et al., 2004

*NR means not relevant. NT means not tested and not suggested in the literature or in this review. Question mark means that the internalization mechanism was suggested in the review or by other authors but not tested. ( ) means that the hypothesized pathway was tested but refued*.

### Secondary Mechanisms

These mechanisms mainly involve bacterial serine aspartate repeat-containing protein D (**SdrD**), clumping factor A (**ClfA**), autolysin (**Atl**), and serine-rich adhesin for platelets **(SraP)**. These proteins are MSCRAMMs and (except for Atl) have the cell-wall anchoring sequence LPXTG located in their C-terminal portion. Their N-terminal part contains a signal peptide for their secretion, followed by a ligand binding region mainly consisting of repeated sequences often rich in Serine. The mechanisms involving these proteins are Fn-independent: SdrD binds directly to Desmoglein 1 on the cell surface of keratinocytes and desquamated nasal cells (Corrigan et al., 2009; Askarian et al., 2016); ClfA interacts directly with host cells or through fibrinogen bridges (McDonnell et al., 2016; Claes et al., 2017); Atl seems to mediate *S. aureus* internalization via direct interactions with Hsc70 (Hirschhausen et al., 2010); and SraP adheres to A549 cells through the salivary scavenger protein gp340 (Yang et al., 2014) (**Figure 1C**). Note that the SdrD and Atl mechanisms require both bacterial and cellular activity as their efficiency depends on the expression of the bacterial and of the cellular interactor. Indeed, SdrD expression is upregulated following contact with neutrophils (Sitkiewicz et al., 2011) and Hsc70 production is stimulated by *S. aureus* infection of EA.hy 926 cells (Hirschhausen et al., 2010). Moreover, Atl has also been shown to be involved in the secretion of several *S. aureus* proteins, among which SdrD (Pasztor et al., 2010). Atl could thus have an “active” role as a secondary mechanism of internalization in some cells and act as a regulator of those secondary mechanisms in other cell types. More generally, these observations might reflect the fact that the bacteria can adapt their internalization strategy to the environmental conditions, i.e., an absence or scarcity of Fn, by finding alternative binding partners and/or by upregulating one side or the other of the adhesion machinery (e.g., SdrD and Atl). For instance, ClfA binds to annexin 2 on the surface of bovine mammary epithelial cells (MAC-T cells) (Ashraf et al., 2017) but has two receptors on the surface of endothelial cells, namely αvβ3 integrin using fibrinogen as a bridge (McDonnell et al., 2016) and von Willebrand factor using self-secreted von Willebrand factor binding protein as a bridge (**Table 1** and **Figure 1C**) (Claes et al., 2017). This might increase its capacity to adhere to and possibly enter endothelial cells, thereby supporting its role in the pathogenesis of endocarditis. Fibrinogen binding also primes platelets aggregation and abscess formation and can lead to thromboembolic lesions in the heart during sepsis (McAdow et al., 2011; Malachowa et al., 2016).

In other cases, the alternative mechanisms may support the FnBP-Fn-α5β1 integrin-mediated uptake of *S. aureus* instead. This is notably illustrated by *S. aureus* extracellular adherence protein (Eap) that plays a role in the adherence to fibroblasts and epithelial cells independently of any binding to Fn or fibrinogen (**Figure 1C**) (Hussain et al., 2002). Recently, its deletion has been shown not to affect the adhesion and internalization steps, contradicting the early idea that its role was to compensate for FnBP function loss (Palma et al., 1999; Grundmeier et al., 2004). Rather, Eap seems to promote the adhesion and internalization of *S. aureus* and other pathogenic bacteria encountered in the context of polymicrobial skin infection (Bur et al., 2013). As for all the previously cited mechanisms, the molecular and signaling pathways underlying this activity are not understood yet and more work is needed to clarify whether Eap (1) is secreted and binds to α5β1 integrin via Fn, triggering the internalization cascade pathway and enhancing the uptake of bacteria bound to molecules other than α5β1 integrin in cells that express this integrin poorly; or (2) triggers actin-dependent phagocytosis, as its effect is fully blocked by cytochalasin D.

The need to elucidate the molecular and signaling pathways of these alternative mechanisms is highlighted by their phenotypical relevance. ClfA and SraP are involved in the pathogenesis of endocarditis. SdrD-mediated binding appears to help the bacteria survive *in vivo* (Askarian et al., 2017)—whether this is because of an increased ability to kill neutrophils and/or to invade cells is an open question. Also, unraveling whether internalization occurs via the same FAK/Src/PI3K/Akt cascade as for the FnBP-Fn-β1 integrin pathway is essential as this may offer a potential avenue to treat chronic infections.

The minor mechanism involving GapC during the adhesion to and invasion of MAC-T cells is also noteworthy as it has been involved in the development of mastitis (Kerro-Dego et al., 2012). Finally, another point to bear in mind is that in addition to adhesins, other (probably less expected) molecules are involved in the adhesion to and invasion of NPPCs by *S. aureus*. In particular, the Tet38 efflux pump extrudes tetracycline and unsaturated free fatty acids but also interacts with CD36, a cellular transporter of long chain fatty acids, to trigger *S. aureus* adhesion and entry into A549 cells (Truong-Bolduc et al., 2017). Tet38 is also involved in phagosomal escape, facilitating the replication and persistence of *S. aureus*.

## Staphylococcus pseudintermedius

*Staphylococcus pseudintermedius* is a coagulase-positive species mostly responsible for infections in dogs, notably necrotic skin lesions and bone and joint infections (Nazarali et al., 2015). However, human infections with *S. pseudintermedius* have also been described, revealing a risk of zoonotic transmission (Darlow et al., 2017; Lozano et al., 2017). Recently, Maali et al. (2016) performed a comparative study examining various CoNS species and reported that *S. pseudintermedius* was the only species that significantly adheres to human Fn and invades MG-63 cells with higher rates than *S. aureus*. This internalization does not occur in β1 integrin-deficient murine osteoblasts and is mediated through SpsD and SpsL, two cell wall-anchored proteins that share homologies with *S. aureus* FnBPA and FnBPB (Pietrocola et al., 2015; Maali et al., 2016).

## *Staphylococcus epidermidis* and Other Coagulase-Negative Staphylococci

The ability of *S. epidermidis* and other CoNS to bind fibronectin and to be internalized has also been investigated. The results of some of these studies and their interpretation are controversial however.

The first study of Fn binding in *S. epidermidis* and other CoNS species found that *S. epidermidis* was a good Fn binder (Switalski et al., 1983). However, this study also revealed huge variations in binding activity between *S. epidermidis* strains and between CoNS species. Later, Minhas et al. (1995) reported the presence of FnBP genes in *S. epidermidis* and other CoNS using PCR inside a repeat unit region (D1–D4) found in both *fnbA* and *fnbB* from *S. aureus* (**Figure 1A**). Still, no FnBP-like protein has clearly been identified in *S. epidermidis* so far and no other study has found FnBPs at genomic, transcriptomic, or protein levels in CoNS. In *S. epidermidis*, the giant extracellular matrix binding protein (Embp) has been shown to bind Fn (Williams et al., 2002). Embp harbors 59 “Found In Various Architectures” (FIVAR) domains, involved in Fn binding (Christner et al., 2010), and 38 protein G-related albumin-binding (GA). A recombinant protein containing the Fn-binding domain of Embp blocks *S. epidermidis* binding to Fn but not that of *S. aureus*. Conversely, the competitive use of recombinant FnBPB (D1–D4 units) has been shown to block the binding of *S. aureus*, but not of *S. epidermidis*, to Fn. *S. epidermidis* Embp and *S. aureus* FnBPs must therefore have distinct Fn interaction sites (Williams et al., 2002).

The internalization of *S. epidermidis* (and that of other CoNS) by NPPCs is a more controversial issue. While several studies have reported that *S. epidermidis* is internalized by different types of NPPCs, namely endothelial cells (Oviedo-Boyso et al., 2009), MAC-T cells (Almeida and Oliver, 2001) and human osteoblast-like MG-63 cells (Khalil et al., 2007), others have minimized its ability to invade MG-63 cells (Valour et al., 2013; Campoccia et al., 2016; Maali et al., 2016). Indeed, using appropriate infection conditions, Valour et al. (2013) and Campoccia et al. (2016) found that even with a multiplicity of infection (MOI) exceeding 500:1, the rate of *S. epidermidis* internalization 2 h after infection was very low. (The rate of *S. epidermidis* internalization at an MOI 500:1 was about 100 times lower than that of *S. aureus* at an MOI of 100:1.) Although the significance of such a low level of invasion by *S. epidermidis* remains unclear, the uptake can happen and the following mechanisms have been proposed and identified to explain this.

One tempting hypothesis is that the internalization of *S. epidermidis* by NPPCs occurs through a tripartite Embp-Fn-α5β1 system analogous to the FnBP-Fn-α5β1 integrin process for *S. aureus*. Using a recombinant protein that blocks the Fn-binding domain of *S. aureus* FnBP, Khalil et al. (2007) completely inhibited the internalization of *S. aureus* but failed to block that of *S. epidermidis*. This fits with there being no FnBP in *S. epidermidis*, as discussed below, but does not refute the “Embp/Fn/α5β1 integrin” hypothesis. However, the use of an anti-α5β1 integrin antibody was found to block the internalization of *S. aureus* but not that of *S. epidermidis* by MG-63 cells (Khalil et al., 2007). The uptake of *S. epidermidis* is therefore independent of the α5β1 integrin, which rules out the “Embp-Fn-α5β1 integrin” system and supports the intervention of secondary mechanisms.

In CoNS, the only internalization mechanism that has been described to date involves Atl. As mentioned above for Atl in *S. aureus, S. epidermidis* is internalized through direct interactions between AtlE and Hsc70 (Hirschhausen et al., 2010). This would seem to be the logical alternative mechanism for internalization as the other CoNS also have autolysins (Albrecht et al., 2012). However, existing studies only describe an ability to bind to Fn through autolysin and/or become internalized but do not offer a mechanistic explanation (**Table 1**) (Hell et al., 1998, 2003; Allignet et al., 1999, 2001, 2002; Szabados et al., 2008; Pereira et al., 2012; Hussain et al., 2015). It is also noteworthy that the involvement in the internalization process of the fibrinogen-binding protein Fbl produced in *S. lugdunensis*, a homolog of *S. aureus* ClfA, has also been investigated, with negative results (Szabados et al., 2011).

## Conclusion

As this short review shows, adhesion to fibronectin is a major explanation for the virulence of staphylococci. In *S. aureus* and *S. pseudintermedius*, adhesion involves fibronectin and α5β1 integrin and leads to internalization in host cells, which favors intracellular persistence and chronic infections. There are alternative mechanisms through which staphylococci become internalized and these may explain why in the absence of a major Fn-related internalization mechanism, *S. epidermidis* still invades cells, albeit at a low level. It is not yet clear whether these secondary mechanisms are completely independent of the FnBP-Fn-α5β1 integrin pathway (i.e., substitute it) or if they support it by strengthening binding or by triggering internalization more effectively. Identifying (1) the detailed molecular mechanisms of the FnBP-Fn-α5β1 pathway, (2) the surface proteins involved in alternative mechanisms, and (3) the role of these mechanisms, would be major steps toward more efficient anti-bacterial treatments for chronic staphylococci infections.

## Author Contributions

JJ and AD prepared the draft of the papers with the help of FL. FL revised the version of the text.

## Conflict of Interest Statement

The authors declare that the research was conducted in the absence of any commercial or financial relationships that could be construed as a potential conflict of interest.
